# Supplementary material for: The NALCN channel regulates metastasis and nonmalignant cell dissemination
Source: Nat Genet. 2022 Sep 29;54(12):1827–38. doi: 10.1038/s41588-022-01182-0 (PMC9729110; doi:10.1038/s41588-022-01182-0)
Supplement: Supplementary file 1 — Supplementary Figs. 1–3. [file 41588_2022_1182_MOESM1_ESM.pdf]

nature portfolio

https://doi.org/10.1038/s41588-022-01182-0

Supplementary information

The NALCN channel regulates metastasis and nonmalignant cell dissemination

In the format provided by the authors and unedited

Supplementary materials for

The NALCN channel regulates metastasis and non-malignant cell dissemination

Eric P. Rahrmann[1], David Shorthouse[2], Amir Jassim[1], Linda P. Hu[1], Mariaestela Ortiz[3], Betania Mahler-Araujo[4], Peter Vogel[5], Marta Paez-Ribes[1], Atefeh Fatemi[1], Gregory J Hannon[1], Radhika Iyer[6], Jay A. Blundon[7], Filipe C. Lourenço[1], Jonathan Kay[8], Rosalynn M. Nazarian[9], Benjamin A. Hall[2], Stanislav S. Zakharenko[7], Douglas J Winton[1], Liqin Zhu[10], Richard J. Gilbertson[1,11*]

Correspondence should be addressed to RJG: Richard.Gilbertson@cruk.cam.ac.uk

This PDF file includes:
Supplementary Figures 1, 2 and 3
Source Data for Supplementary Fig. 2

Reagents, antibodies and software

A table of the source of all reagents, antibodies, kits, cell lines, chemical and software are included (Table SX)

Supplementary Figures, Tables:

Fig. S1. Single channel immunofluorescent images for all figures and extended data figures.

Fig. S2. CZC standard curves generated by two 'spike-in' control techniques.

Fig. S3. Supplementary Figure S3. Exemplar FACS gating strategies for isolating

ZSG+ cells from peripher-al blood samples.

Source data for Supplementary Figure S2.

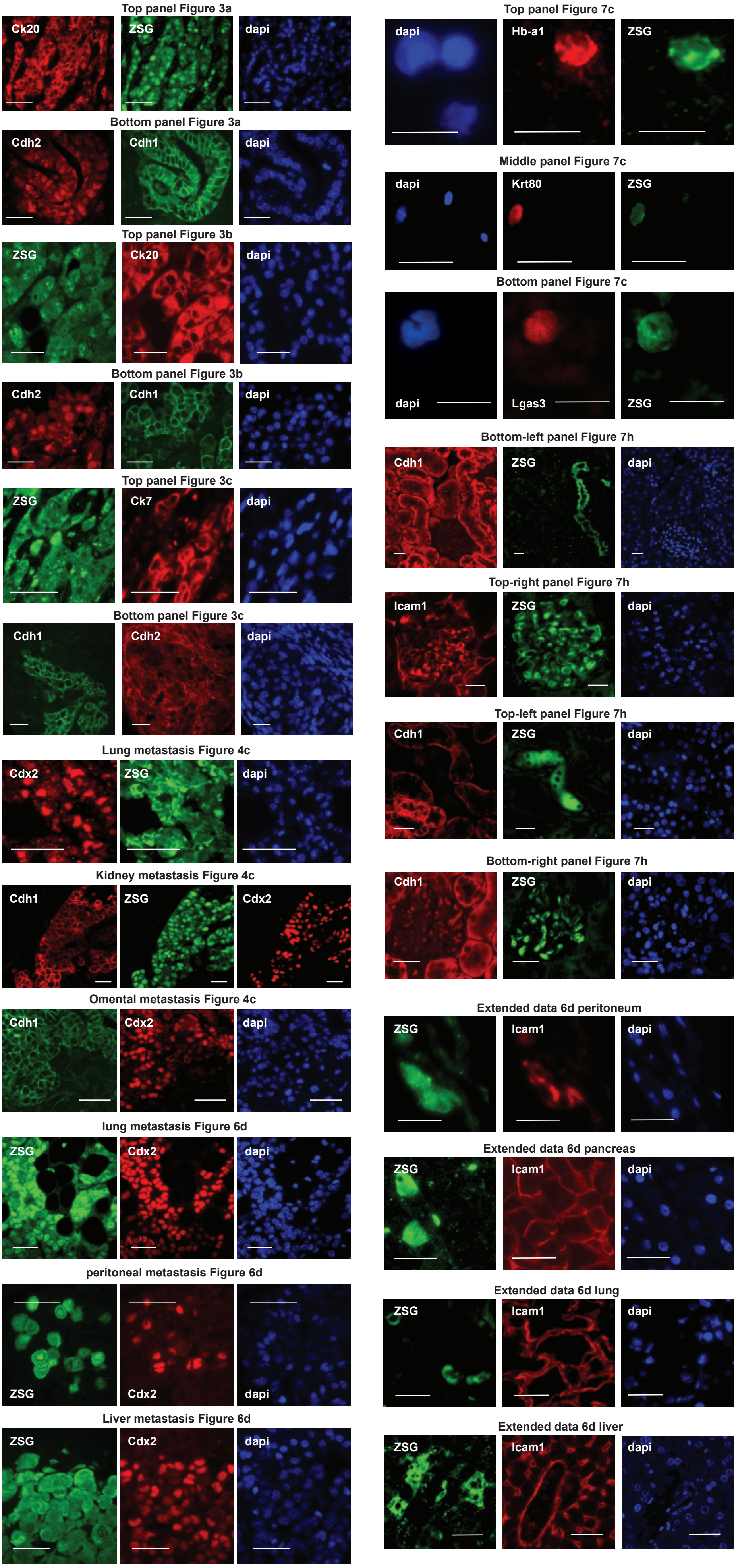

Supplementary Information Figure 1: single channel images of immunofluorescence studies of the indicated panels from main and extended data. All scale bars=50μm except for Figure 7c that are 10μm.

Supplementary Figure 2

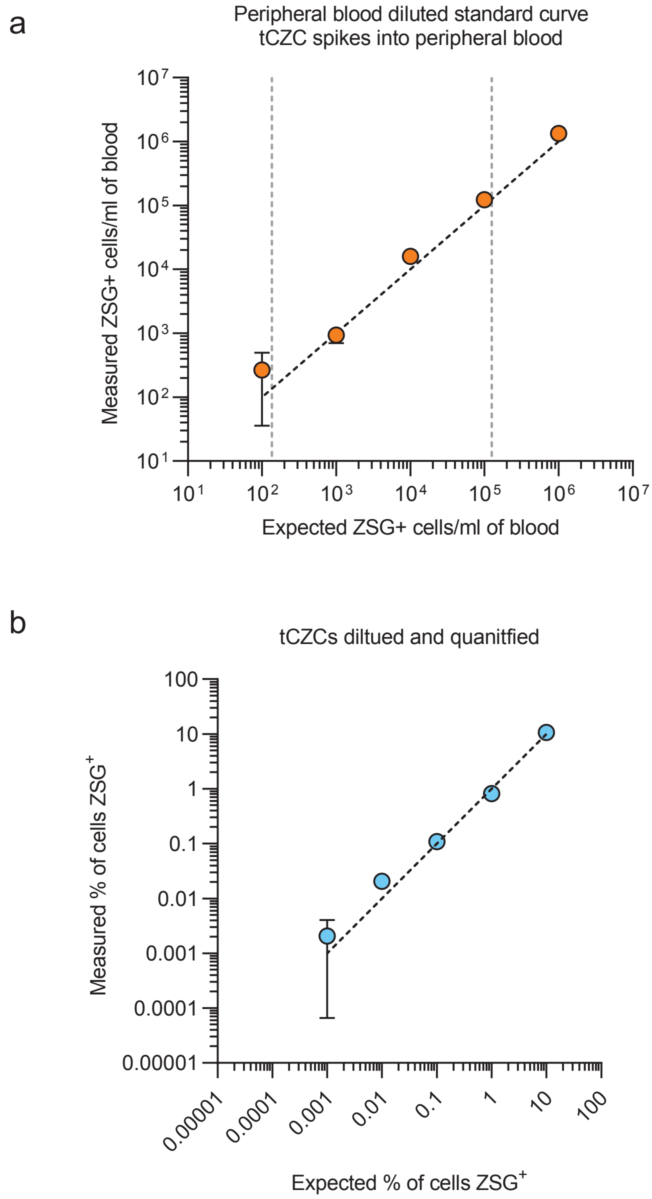

Standard curves generated by two 'spike-in' control techniques. (a) Normal peripheral blood was harvested from adult *P1-KP* mice with gastric adenocarcinoma. Peripheral blood mononuclear cells (PBMCs) were isolated by ficoll gradient centrifugation. ZSG^+ cells were enumerated manually and spiked into fresh peripheral blood to give the final number of actual ZSG+ cells/ml (x-axis). These samples were then subject to the same FACS protocol used in all blood isolation studies to provide the observed (y-axis) quantification. The vertical dotted lines represent the 25th and 75th percentile of observed CZCs/ml recorded in Supplementary Table 11. **(b)** Normal PBMCs used in (a) were also spiked at the indicated percentage of total PBMCs in buffered saline and quantified in the same manner as in (a). In both graphs, the hashed black line represents the ideal curve in which expected and observed results are the same.

Supplementary Figure 3

a

tCZC example 1: ZSGreen negative

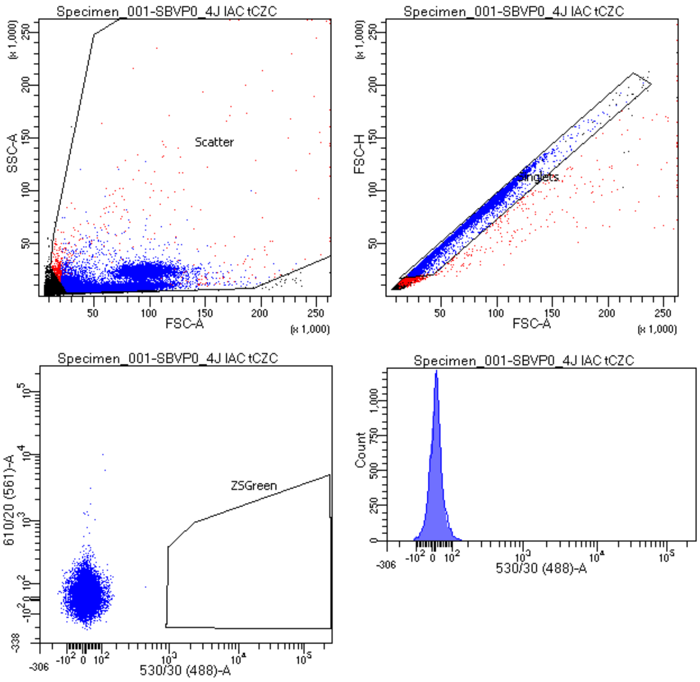

Tube: SBVP0_4J IAC tCZC			
Population	#Events	%Parent	%Total
All Events	100,000	####	100.0
Scatter	13,316	13.3	13.3
Singlets	12,624	94.8	12.6
ZSGreen	0	0.0	0.0

b

tCZC example 2: ZSGreen positive

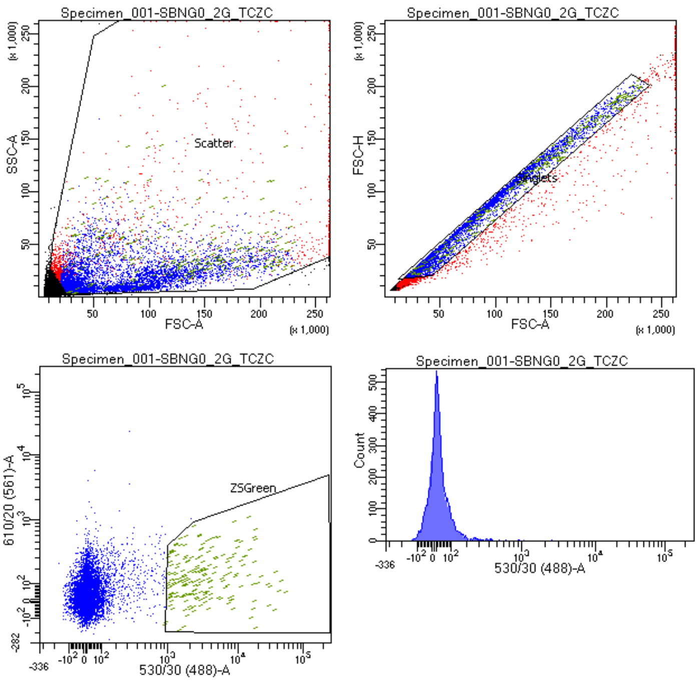

Tube: SBNG0_2G_TCZC			
Population	#Events	%Parent	%Total
All Events	50,000	####	100.0
Scatter	8,012	16.0	16.0
Singlets	6,685	83.4	13.4
ZSGreen	191	2.9	0.4

Supplementary Figure S3. Exemplar FACS gating strategies for isolating ZSG+ cells from peripheral blood samples. (a) Example of a tumour-bearing animal that did not have ZSG+ cells in the peripheral blood. (b) Example of a tumour-bearing animal with ZSG+ cells in the the peripheral blood.

Source data for Supplementary Fig. S2a and b.

Expected # of ZSG+ cells/ml blood	GAC (*Nalcn*$^{flx/+}$) 1	GAC (*Nalcn*$^{flx/+}$) 2	GAC (*Nalcn*$^{flx/+}$) 3
1000000	**1392000**	**1356000**	**1244000**
100000	**148800**	**107600**	**110400**
10000	**13960**	**16000**	**17640**
1000	**800**	**800**	**1200**
100	**400**	**0.001**	**400**

Expected % of ZSG+ cells/ml blood	GAC (*Nalcn*$^{flx/+}$) 1	GAC (*Nalcn*$^{flx/+}$) 2	GAC (*Nalcn*$^{flx/+}$) 3
10	**10.4467**	**9.2553**	**12.0833**
1	**0.79**	**0.8013**	**0.8498**
0.1	**0.0992**	**0.0867**	**0.1404**
0.01	**0.0167**	**0.0213**	**0.0234**
0.001	**0.002**	**0.0041**	**0.0001**
