# Supplementary material for: The NALCN channel regulates metastasis and nonmalignant cell dissemination
Source: Nat Genet. 2022 Sep 29;54(12):1827–38. doi: 10.1038/s41588-022-01182-0 (PMC9729110; doi:10.1038/s41588-022-01182-0)
Supplement: Supplementary file 2 — Reporting Summary [file 41588_2022_1182_MOESM2_ESM.pdf]

nature portfolio

Corresponding author(s): Richard J Gilbertson

Last updated by author(s): June 17th 2022

Reporting Summary

Nature Portfolio wishes to improve the reproducibility of the work that we publish. This form provides structure for consistency and transparency in reporting. For further information on Nature Portfolio policies, see our Editorial Policies and the Editorial Policy Checklist.

Statistics

For all statistical analyses, confirm that the following items are present in the figure legend, table legend, main text, or Methods section.

n/a	Confirmed	
☐	☒	The exact sample size (*n*) for each experimental group/condition, given as a discrete number and unit of measurement
☐	☒	A statement on whether measurements were taken from distinct samples or whether the same sample was measured repeatedly
☐	☒	The statistical test(s) used AND whether they are one- or two-sided<br>*Only common tests should be described solely by name; describe more complex techniques in the Methods section.*
☐	☒	A description of all covariates tested
☒	☐	A description of any assumptions or corrections, such as tests of normality and adjustment for multiple comparisons
☐	☒	A full description of the statistical parameters including central tendency (e.g. means) or other basic estimates (e.g. regression coefficient) AND variation (e.g. standard deviation) or associated estimates of uncertainty (e.g. confidence intervals)
☐	☒	For null hypothesis testing, the test statistic (e.g. *F*, *t*, *r*) with confidence intervals, effect sizes, degrees of freedom and *P* value noted<br>*Give P values as exact values whenever suitable.*
☒	☐	For Bayesian analysis, information on the choice of priors and Markov chain Monte Carlo settings
☐	☒	For hierarchical and complex designs, identification of the appropriate level for tests and full reporting of outcomes
☒	☐	Estimates of effect sizes (e.g. Cohen's *d*, Pearson's *r*), indicating how they were calculated

Our web collection on statistics for biologists contains articles on many of the points above.

Software and code

Policy information about availability of computer code

Data collection	MACSQuant software (version 10), Microsoft Excel (version 16.49), TCGA via cBioportal, Xenabrowser, HALO v2.0
Data analysis	No custom code was used for any part of the data processing or analysis. Log-Rank (Mantel-Cox) test, Mann Whitney U two-tailed tests and multi-variate analyses were performed using Prism (version 10). RNAsequencing data (human and mouse, bulk and single cell) and dN/dS were done using R software (3.6.1), R studio (1.3.1093),and python (3.9) (see methods for details). g:GOSt for gene enrichment analysis by cumulative hypergeometric probability and multiple testing correction. MORPHEUS for pair-wise comparison analyses and hierarchical clustering, https://software.broadinstitute.org/morpheus. Code used for analyses can be found here: https://github.com/shorthouse-mrc/NALCN

For manuscripts utilizing custom algorithms or software that are central to the research but not yet described in published literature, software must be made available to editors and reviewers. We strongly encourage code deposition in a community repository (e.g. GitHub). See the Nature Portfolio guidelines for submitting code & software for further information.

Data

Policy information about availability of data

All manuscripts must include a data availability statement. This statement should provide the following information, where applicable:

- Accession codes, unique identifiers, or web links for publicly available datasets
- A description of any restrictions on data availability
- For clinical datasets or third party data, please ensure that the statement adheres to our policy

All the raw sequencing data have been deposited in the Gene Expression Omnibus with the following accession numbers: mouse RNA-seq of tumours and

metastases (GSE210134) and mouse single cell RNA-seq of CZCs, PBMCs, tumours, metastases and solid tissues (GSE210134). Murine Prom1+ gastric mucosa and adenocarcinoma data GEO accession number: GSE78076. NALCN mutation and TSNE plot were generated with Pan-Cancer Atlas data from TCGA via cbioPortal and Xenabrowser. Cancer staging data were generated with Pan-Caner Atlas from TCGA and COSMIC data. NALCN structure 6XIW was from pdb. Human CTC and gene signature datasets are from the following GEO accession numbers: GSE75367, GSE74639, GSE60407, GSE67980, GSE114704, GSE144494. Human PBMC data are from Illumina 10X (10k Human PBMCs, 3' v3.1, Chromium X). Source data are provided with this paper.

Field-specific reporting

Please select the one below that is the best fit for your research. If you are not sure, read the appropriate sections before making your selection.

☒ Life sciences ☐ Behavioural & social sciences ☐ Ecological, evolutionary & environmental sciences

For a reference copy of the document with all sections, see nature.com/documents/nr-reporting-summary-flat.pdf

Life sciences study design

All studies must disclose on these points even when the disclosure is negative.

Sample size

Sample sizes for each experiment are indicated in the figures, figure legends or main text. No statistical methods were used to predetermine sample size. The sample sizes for animal experiments were chosen based on previous experience in the lab and from published works specifically on tumour penetrance and latency. Zhu et al 2016, Jackstadt et al 2019, Hingorani et al 2003. Samples from single cell RNAseq generated thousands of cells which were acquired across several different genetic cohorts. Aside from animal experiments, we executed a minimum of three biological replicates whether it be for in vitro cell culture based assays, histological analyses, or transciptomic analyses. In some cases where only three biological replicates were done, it was dictated by availability of materials.

Data exclusions

Cell selection and filtering criteria for the single cell RNAseq analyses are detailed in the Methods section. Quality control metrics were generated using Scater. We removed cells on a per sample basis which had greater than 3 MADs (median absolute deviations) above the mean percentage of mitochondrial content, cells which had greater or fewer genes detected than 3 MADs above or below the mean and cells which had greater or fewer UMIs than 3 MADs above or below the mean. Genes which were detected in less than 5 cells (across all samples) were then removed from the matrix. Single cell objects filtered according to criteria above were generated from this step. Single cell objects were then converted to Seurat objects using the as.Seurat function. Doublet and negative cells were removed from objects representing solid organs (hash-tagged data). Multiple Seurat objects representing different samples were merged together using the merge function before a universal filtering step was carried out. Cells were filtered based on a threshold of cells with more than 100 and less than 5000 genes and less than 20% mitochondrial content.

Replication

All experiments were performed in at least 3 biologically independent replicates. All replicates on which statistics were carried out are biological replicates. In the case of genetically engineered mouse models, studies were balanced for age and sex. For the single cell RNAseq, cell types were reliably identified across multiple biological replicates. All attempts at replication of the results were successful.

Randomization

For in vivo work, induction of genetic alterations occurred either spontaneously embryonically or induced with tamoxifen exposure at postnatal day 3 or 60. Animals were chosen based on correct genotypes based on presence of a cre-recombinase allele (Nestin-cre, Pdx1-cre, villin1-CreERT2, Prom1-CreERT2, Rosa26-CreERT2), a reporter allele (Rosa26-ZSGreen), one of 3 combinations of Nalcn allele (wt, het, null) and then optionally combinations of oncogenic alleles for tumour studies (KrasG12D, Trp53flx). Sex-specific differences were minimized by including similar number of male and female animals. Each experiment contained animals from several different litters over the course of 2 years. For aging tumour studies, randomization was not applicable. For the gadolinium induciton experiments, all littermates were injected with gadolinium to ensure we captured representation of all Nalcn allele combinations.
In allograft experiments we utilized female mice from 4-16 weeks of age (NSG, Foxn1) that were purchased from Charles River. Age range was due to availability of primary material (circulating ZSG+ cells) from donor animals. Again, randomization was not applicable for these studies.

For studies where gastric spheres were expose to 4-hydroxytamoxifen, 8 wells of a 12-well plate were seeded with syngenic gastric organoids. 4 wells were chosen on the plate to receive 4-hydroxytamoxifen which in which 2 wells from each row were choosen to balance plating positioning effects.

Blinding

Multiple investigators were involved with sample collection, annotation, observational recordings and analysis and all were blinded to animal genotypes. For gastric sphere assays counting, electrophysiology researchers were blinded to the conditions for data collection and image analysis. For blood sample analyses, at time of blood draw only the animal ID was known and nothing about the genotype. Samples were analyzed and data collected prior to de-identifying. For metastasis enumeration, all data were collected without knowledge of the animal genotype.

Reporting for specific materials, systems and methods

We require information from authors about some types of materials, experimental systems and methods used in many studies. Here, indicate whether each material, system or method listed is relevant to your study. If you are not sure if a list item applies to your research, read the appropriate section before selecting a response.

Materials & experimental systems

n/a	Involved in the study
☐	☒ Antibodies
☐	☒ Eukaryotic cell lines
☒	☐ Palaeontology and archaeology
☐	☒ Animals and other organisms
☒	☐ Human research participants
☒	☐ Clinical data
☒	☐ Dual use research of concern

Methods

n/a	Involved in the study
☒	☐ ChIP-seq
☐	☒ Flow cytometry
☒	☐ MRI-based neuroimaging

Antibodies

Antibodies used

For immunofluorescence and immunohistochemistry the following antibodies were used: Rhodamine-labeled DBA (RL-1032, Vector Laboratories, 1:100), rhodamine-labeled UEA I (RL-1062, Vector Laboratories, 1:100), ZSGreen (mouse monoclonal, TA180002, Origene, 1:1000), CD31 (rabbit polyclonal, 77699, Cell Signaling Tehcnology, 1:100), CK7 (rabbit monoclonal [EPR17078],ab181598, Abcam, 1:200), CK20 (rabbit monoclonal,ab97511, Abcam, 1:200), E-cadherin (goat polyclonal, AF748, R&D systems, 1:100), N-cadherin (rabbit monoclonal, 13116, Cell Signalling Technology, 1:100), Icam1 (rabbit monoclonal, ab179707, Abcam, 1:100), Cdx2 (rabbit monoclonal, ab76541, Abcam, 1:100), aSMA (rabbit polyclonal, ab5694; Abcam; 1:500), Krt80 (rabbit polyclonal, 16835-1-AP, ProteinTech, 1:100), Hba-a1 (rabbit monoclonal, ab92492, Abcam, 1:100), Galectin3 (Lgals3) (rabbit monoclonal, ab209344, Abcam, 1:200), CD45 (rabbit polyclonal, ab10558, Abcam, 1:200), CD45 (ab25386, Abcam), Cleaved Caspase 3 (9664, Cell Signaling Technology, 1:200), Ki67 (IHC-00375, Bethyl, 1:1000). Following washing, tissue sections where then incubated for 1 hour at room temperature in secondary antibody. Secondary antibodies included Alexa 488, 594, 647 (A-11055, A-21207, A-31571, ThermoFisher, 1:500) of polymer based reagents (Leica Plymer Refine Detection System (DS9800, Leica biosystems) and Rabbit anti-rat polymer (A110-322a, Bethyl Laboratories, 1:250). Sections were then counterstained using DAPI (4083; Cell Signaling, 1:10,000) or Hematoxylin.

Validation

Abcam antibodies that are KO validated by the distributor: Lgals3 (rabbit monoclonal, ab209344, Abcam, 1:200). No knockout validation was carried out for any remaining antibodies..All other validation was done on primary tissues where the expected profile of the antibody was negative (e.g. Cdx2 expressed in GI tract but not lymphoid tissue) and also ensuring the correct compartmentalization of the antibodies (e.g. Cdh1 membrane bound) For over, for each of the antibodies when first using we would carryout no primary control histology to identify any background staining patterns and compared to slides that received the primary antibody.

Eukaryotic cell lines

Policy information about cell lines

Cell line source(s)

Primary mouse gastric spheres generated from P1-KP gastric adenocarcinomas and P1-N normal gastric tissue., 293FT, L wnt3a

Authentication

None of the primary cell lines have been authenticated. The 293FT and L wnt3a were puchased from Thermo Fisher and ATCC, respectivley.

Mycoplasma contamination

The cell lines were tested prior to implantation into recipient immunocompromised animals and were negative.

Commonly misidentified lines
(See ICLAC register)

None of the cell lines used are listed in the databased of commonly misidentified cell lines maintained by ICLAC.

Animals and other organisms

Policy information about studies involving animals; ARRIVE guidelines recommended for reporting animal research

Laboratory animals

All genetically engineered mouse models were mixed background. Animals carrying the modified Nalcn allele were bred to RosaFLPe expressing mice to remove LacZ and Neo cassette. Animals with complete recombination were used for subsequent breeding combinations of various previously described strains: Prom1C-L; Nestin-cre; Rosa-CreERT2; villin-CreER; Pdx1-cre; RosaZSG; KrasG12D/+, Trp53flx were interbred and maintained on mixed Bl6/FVBN backgrounds. Both males and females were used in these experiments. Induction of recombination occurred either spontaneously embryonically (Nestin-cre, Pdx1-cre) or were induced postnatally (day 3 or day 60) with tamoxifen and aged up to 2 years. For grafting experiments NSG/Foxn1nu/nu mice were used with age ranging from 4- 16 weeks. Female mice were only used in these experiments. Mice were housed in individually ventilated cages with wood chip bedding and nestlets with environmental enrichment (cardboard fun tunnels and chew blocks) in 12 h light/dark cycle at 21 ± 2 °C, humidity = 55% ± 10%. Diet was irradiated LabDiet 5R58 with ad libitum water.

Wild animals

study did not involve wild animals

Field-collected samples

study did not involve samples collected from the field

Ethics oversight

All animal studies within the United Kingdom were performed under the Animals (Scientific Procedures) Act 1986 in accordance with UK Home Office licenses (Project License 70-8823, P47AE7E47, PP7834816) and approved by the Cancer Research UK (CRUK)

Cambridge Institute Animal Welfare and Ethical Review Board. All studies conducted at St. Jude Children's Research Hospital were approved by the Institutional Animal care and Use Committee of St. Jude Children’s Research Hospital.

Note that full information on the approval of the study protocol must also be provided in the manuscript.

Flow Cytometry

Plots

Confirm that:

☒ The axis labels state the marker and fluorochrome used (e.g. CD4-FITC).

☒ The axis scales are clearly visible. Include numbers along axes only for bottom left plot of group (a 'group' is an analysis of identical markers).

☐ All plots are contour plots with outliers or pseudocolor plots.

☒ A numerical value for number of cells or percentage (with statistics) is provided.

Methodology

Sample preparation

Peripheral blood (500ml-1ml) was harvested from mice at necropsy, 10ul of 0.5M EDTA was added to the blood to prevent clotting and subject to red blood cell lysis (420301, BioLegend). ZSGreen cells were quantified using MACSQuant Analyzer 10 Flow cytometer (Miltenyi biotech). ZSGreen+ (ZSG+) sorted and quantified using a BD FACSAria II Cell Sorter (BD Biosciences) and the BD Influx Cell Sorter (BD Biosciences) with excitation at 525/50nm (FITC) vs 614/50nm (PI). Non-tamoxifen induced mouse peripheral blood served as a negative control to set parameters.

Instrument

MACSQuant Analyzer 10 Flow cytometer (Miltenyi biotech)
BD FACSAria II Cell Sorter (BD Biosciences)
BD Influx Cell Sorter (BD Biosciences)

Software

MACSQuant (Miltenyi biotech) and FACSAria Software (BD Biosciences)

Cell population abundance

ZSGreen populations range from 0 events up to 10% of events across all cell populations assessed.

Gating strategy

Living cells were selected by forward scatter and side scatter. ZSGreen positive populations were determined by using tamoxifen induced animal tissues as negative controls to set parameters.

☒ Tick this box to confirm that a figure exemplifying the gating strategy is provided in the Supplementary Information.
