# Supplementary material for: The NALCN channel regulates metastasis and nonmalignant cell dissemination
Source: Nat Genet. 2022 Sep 29;54(12):1827–38. doi: 10.1038/s41588-022-01182-0 (PMC9729110; doi:10.1038/s41588-022-01182-0)
Supplement: Supplementary file 12 — Unprocessed gel. [file 41588_2022_1182_MOESM12_ESM.pdf]

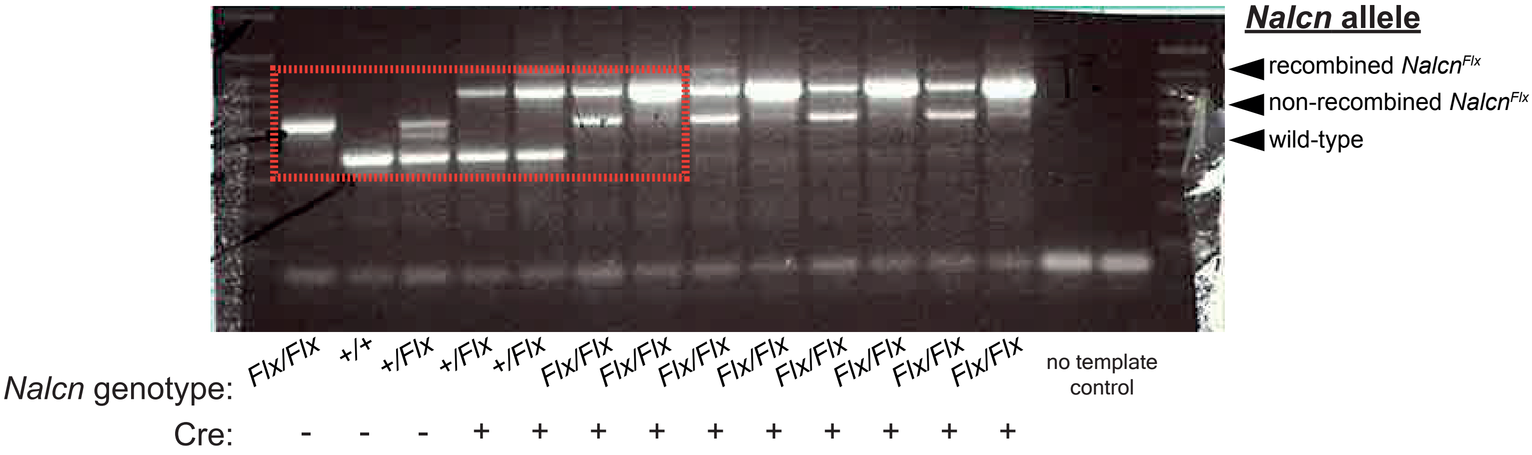

Source data EXTENDED DATA FIG 2A: uncropped gel image of Extended Data FIG 2a. Polymerase chain reaction (PCR) products derived from brains of *Nalcn*$^{+/+}$, *Nalcn*$^{+/Flx}$, or *Nalcn*$^{Flx/Flx}$ mice with or without the NestinCre allele. 1% agarose gel was used to resolve bands with a 100bp DNA ladder as reference point. wild-type band 389bp, non-recombined *Nalcn*^{Flx} band 494bp, recombined *Nalcn*^{Flx} band 651bp. Area cropped for Extended Data FIG 3a higlighted with dashed box in red.
